# Pulmonary Tuberculosis-its Pathology, Nature, Symptoms, Diagnosis, Etc.

**Published:** 1875-03-15

**Authors:** 


PULMONARY TUBERCULOSIS—ITS PATHOLOGY, NATURE, SYMPTOMS, DIAGNOSIS, ETC. By Addison P. Dutcher, M.D. Philadelphia: J. B. Lippincott & Co. For sale by Keen, Cooke & Co., Chicago.

This is a volume of 380 pages, and forms a very complete, comprehensive and practical treatise on the subject of pulmonary tuberculosis. The author does not put forward any especially new theories regarding either the pathology or treatment of the disease, but gives a fair, clear statement of all the recognized doctrines, and of the latest studies and investigations of the subject.

In considering the nature of pulmonary tuberculosis, the various theories are briefly reviewed under the headings of inflammatory, imperfect nutrition, defective respiration, specific morbid condition of the blood, imperfect innervation, and retrograde metamorphosis of the tissues. In summing up his own conclusions, the author says: "From a careful review of the whole subject, we think we are fully warranted in drawing the following conclusions:

1st. "That pulmonary tuberculosis is a specific disease, depending upon a morbid condition of the blood, which leads to a discharge of some of its depraved constituents on the external surface of the air cells, and under the basement membrane.

2d. "That this morbid matter is not capable of being assimilated into their textures, nor in any way contributing to their growth or maintenance, and ultimately leads to their dissolution and all its attendant phenomena.

3d. "That the tubercular diathesis increases, and is attended with cachexia, which is often disproportionate to the local disorder, thus early proving its specific and constitutional origin. Tubercular disease may, however, sometimes, cease in part; yet, if we look to its fearful mortality as an index of its natural course, we may see in it a law of increase like that exemplified in some of the more malignant disorders, such as cancer. And such a law is not exemplified in ordinary local diseases, for they generally tend to subside with the lapse of time."

These conclusions are fairly stated, and embody, we think, the now most generally recognized doctrine of the nature of tubercular disease.

In succeeding chapters, the condition of the blood and of the various organs in pulmonary tuberculosis, and the general symptoms, physical diagnosis, etc., of the disease are considered.

Among the physical signs, the author gives more prominence than most writers on the subject, to the dark red or purplish margin along the edge of the gums, which is denominated Thompson's gingival margin. This he considers one of the earliest, most constant, and positive evidences of the tubercular diathesis. He considers it of especial value as one of the few signs diagnostic of the early or pre-tubercular stage of the disease; where, if recognized, it is comparatively manageable and amenable to treatment.

The various symptoms and complications which are common to the different stages of pulmonary tuberculosis, are discussed under appropriate headings. Numerous cases from the author's abundant experience, illustrative of peculiarities in pathology, diagnosis or treatment, are also interspersed throughout the work.

The causes of phthisis, are discussed under two heads: the hereditary and the accidental. Among the latter, variations of temperature, imperfect nutrition, depressing and exciting passions, intemperance, etc. are classed as the most prominent. The possibility of transmission by inoculation or contagion, is also briefly considered. Several chapters are devoted to food, clothing, exercise, and various hygienic regulations for the preservation and restoration of health of consumptives, with a last closing chapter on alcoholic stimulants. The chemical nature and the physiological action of alcohol are considered, and the theories of those who advocate their use in pulmonary tuberculosis are fairly stated. The author gives the weight of his influence and opinion with that of many other careful observers, as opposed to their use, and denying entirely their value or efficiency, in either arresting or retarding the progress of pulmonary tuberculosis.

There is running through the work a vein of sentiment and religious fervor, breaking forth occasionally into poetical rhapsody—passages, beautiful, eloquent and appropriate in themselves, judged from a literary standpoint, but which strike one as a little odd or misplaced in a plain, matter-of-fact, scientific treatise.

As a whole, however, we heartily and conscientiously endorse each and all of the views and opinions advanced by the author.

